# Medicine Taking

**Published:** 1893-07

**Authors:** John E. White


### MEDICINE TAKING.

Medical practice is greatly debased by the less worthy of its professors, but the public are also to blame for much of its errors. Whether as a natural result of eagerness to see *something done* for the relief of their sick friends, or as a consequence of habits handed down from ignorant times, there is a general prejudice against all practice which does not involve a liberal exhibition of medicine. It must of course often be that only a careful study of the case, directions for the proper care of the patient, and a supervison of the treatment which he receives, is all that is properly required of a medical man. Medicines may not be required, or may be calculated to produce injurious effects, even in the smallest quantities.

But when the medical man finds that procedure such as *he* believes to be necessary is unfavorably regarded by those who call him in, and that if he persists in it, they will discharge him and call another, he is apt to give way, and order a few medicines such as he believes may do the least possible harm.

He ought not to take this course; but the temptation is strong, and a regard to his own interest probably carries the day. Thus the practice of medicine is vitiated, the minds of practitioners are depraved, and the character of the whole profession is lowered. Thus a prejudice is found, to the effect that from illness of any kind medicine is inseparable, and an American is very apt to take powders and pills on the slightest experience of an unpleasant sensation, or perhaps no sensation of the kind, but only to prevent illness. Accordingly, an enormous amount of medicine is consumed needlessly in America. All over the land there are pill warehouses like castles. Large fortunes are realized by patent medicines of the most doubtful character; and the public health is by these means undoubtedly much injured.

Of a great many anecdotes told to me by one well acquainted with medical practice, I shall select one as an illustration of the extent of prejudice existing upon this subject, and its effects in corrupting practitioners. An elderly lady had received a hurt in her arm, which required the attendance of a medical practitioner residing at two or three miles distance. He dressed it about twenty times, and saw it completely healed. Now was his time to consider how he should be paid. "My only chance," said he to himself, "is to begin ordering medicine." He therefore affected to think unfavorably of the appearance of the skin of her arm; it betokened a bad state of blood. "I shall send you something for it," said he. He now began a course of medicine to which the old lady very willingly submitted, and at length when it amounted to fifty dollars, he admitted she was well and sent in his bill. When he next called, she told him she had got the bill and was wishing to pay it. "But I think," said she, "you must have surely committed a mistake in drawing it." "What seems wrong, ma'am?" inquired he. "If there be any error, of course we can easily rectify it."

"Oh, why, you have forty dollars here for medicines—that is all very well—I have had *that*. But here you have ten dollars for dressing my arm. Now, you know, I had nothing there, you were only put to a little trouble, which was the same as nothing. I cannot understand this part of your bill at all."

"Oh, very well," said he, "if you think so we'll deduct the charge for dressing." It is needless to add that the balance was ample remuneration for his services as well as his medicines.

It is well to remember, that in a vast number of cases of illness, the only thing required is right disposal and treatment of the patient, for the direction of which is as necessary as for the dispensation of therapeutics.

This skill has cost its possessor much time and money; it is therefore as well entitled to its reward when only employed in giving needful directions, as when prescribing medicines. Let no one suppose that a medical attendant is doing nothing when he does not dose, or give a great many orders.

He often does his duty best by doing nothing; and even for this, supposing him to act with judgment and conscientiousness, he is fully entitled to his remuneration.

JOHN E. WHITE.

